# Dynamic Interspinous Process Distance and Adjacent Vertebral Fracture After Balloon Kyphoplasty: Preliminary Evidence From a Single-Center Cohort

**DOI:** 10.7759/cureus.93578

**Published:** 2025-09-30

**Authors:** Masakazu Toi, Keishi Maruo, Fumihiro Arizumi, Kazuya Kishima, Mitsuhiro Nishizawa, Marika G Rosenfeld, Toshiya Tachibana

**Affiliations:** 1 Department of Orthopaedic Surgery, Hyogo Medical University, Nishinomiya, JPN; 2 Department of Orthopaedic Surgery, Miyoshi Hospital, Nishinomiya, JPN; 3 Department of Orthopaedic Surgery, Orthopaedic Trauma Institute (OTI), University of California, San Francisco (UCSF), San Francisco , USA; 4 Department of Orthopaedic Surgery, Orthopaedic Trauma Institute (OTI), University of California, San Francisco (UCSF), San Francisco, USA

**Keywords:** adjacent vertebral fracture, balloon kyphoplasty, interspinous process distance, osteoporotic vertebral fracture, posterior spinal instability

## Abstract

Purpose: The aim of this study was to investigate whether dynamic changes in interspinous process distance (ΔISPD) between standing and supine positions predict adjacent vertebral fractures (AVFs) following balloon kyphoplasty (BKP) for thoracolumbar osteoporotic vertebral fractures (OVFs). We hypothesized that greater ΔISPD reflects posterior instability and is associated with a higher risk of AVF.

Overview of literature: AVF is a common complication after BKP. Risk factors such as low bone mineral density and endplate injury have been identified, but little attention has been paid to dynamic posterior instability as evaluated by ΔISPD.

Methods: This retrospective observational exploratory pilot study included 36 patients (mean age, 81.8 years) who underwent BKP for thoracolumbar OVFs between 2019 and 2023. ISPD was measured at levels adjacent to the fractured vertebra in both standing and supine lateral radiographs. ΔISPD was normalized to anterior vertebral body height and multiplied by 100 for analysis. Interobserver and intraobserver reliability was assessed. Univariate analyses were conducted using the Mann-Whitney U test or Fisher’s exact test, as appropriate. Receiver operating characteristic (ROC) curve analyses were performed to determine optimal cutoff values for ΔISPD and normalized ΔISPD associated with AVF occurrence.

Results: AVFs occurred in 12 patients (33%). Normalized ΔISPD was significantly associated with AVF occurrence (p < 0.05). These thresholds corresponded to physical ISPD changes of ≥3.4 mm and ≥5.5 mm, respectively. ROC analysis demonstrated moderate predictive performance with area under the curve (AUC) values of 0.750 (upper, 95% CI, 0.58-0.93) and 0.759 (lower, 95% CI, 0.57-0.95).

Conclusions: Dynamic ISPD changes were significantly associated with AVF after BKP. Although this study was limited by the small sample size and event count, normalized ΔISPD appears to be a reproducible and easily obtainable radiographic marker that may aid in identifying patients at high risk for AVF. Incorporating ΔISPD into preoperative assessment could improve risk stratification, but prospective multicenter validation is warranted.

## Introduction

Osteoporotic vertebral fractures (OVFs) represent a growing clinical challenge in aging societies, as they often result in back pain, progressive kyphosis, and reduced activities of daily living (ADLs). These consequences contribute to decreased quality of life and shortened healthy life expectancy in older adults. As such, both the prevention of fractures and the implementation of effective post-fracture interventions are critical. Given the frailty of elderly patients, minimally invasive procedures are particularly preferred.

Balloon kyphoplasty (BKP) has gained widespread use as a minimally invasive technique for managing OVFs. It offers rapid pain relief and improved quality of life by stabilizing the vertebral body and restoring height [1-3]. However, notable postoperative complications of BKP include the development of adjacent vertebral fractures (AVFs), which occur in approximately 4% to 34% of cases [4,5]. AVFs can lead to recurrent pain and may necessitate additional surgical interventions, making them a clinically important issue. Although previous studies have identified several risk factors for AVF, including low bone mineral density, endplate violation, intradiscal cement leakage, and excessive correction angles [6,7], few have examined posterior spinal instability as a potential contributor. The interspinous process distance (ISPD) has been used to assess posterior element instability or pseudoarthrosis, particularly in the cervical spine [8,9], but its relevance in thoracolumbar pathology remains largely unexplored. Therefore, this study aimed to investigate whether dynamic changes in ISPD between standing and supine positions (ΔISPD) are associated with AVF occurrence following BKP for thoracolumbar OVFs.

## Materials and methods

Study design and participants

This retrospective observational study included 36 patients who underwent BKP for OVF localized to the thoracolumbar junction at our institution between 2019 and 2023. All patients had a minimum follow-up period of 12 months. The mean age was 81.8 years, with 28 female and 8 male patients. The indication for BKP was based on the criteria described by Takahashi et al. [10,11], which included a high risk of pseudarthrosis characterized by diffuse low signal on T1-weighted MRI and either localized high or diffuse low signal on T2-weighted MRI. Exclusion criteria were metastatic spinal tumors, spinal infections, and a history of spinal instrumentation surgery. This study was approved by the institutional review board (approval number: 4054).

Imaging evaluation and ISPD measurement

**Figure 1:**
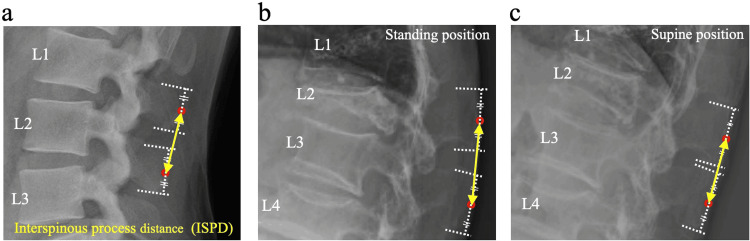
Radiographic measurement of interspinous process distance (ISPD). (a) Definition of ISPD as the linear distance between the midpoints of adjacent spinous processes on lateral radiographs. (b) Standing position and (c) supine position radiographs of a representative case with an L2 osteoporotic vertebral fracture, used for ΔISPD calculation. Digitally drawn open circles indicate the midpoint of each spinous process used for ISPD measurement in panels (b) and (c). Measurements were performed on 150% magnified images to improve reliability as previously described for cervical ISPD. ΔISPD was defined as standing ISPD minus supine ISPD; normalized ΔISPD was ΔISPD divided by L4 anterior vertebral height multiplied by 100.

The ISPD was defined as the linear distance between the midpoints of adjacent spinous processes on lateral radiographs obtained in standing and supine positions. For each spinous process, two tangent lines parallel to the superior endplate of the corresponding vertebral body were drawn at the cranial and caudal tips, and the midpoint was defined as the halfway point along the perpendicular line connecting these tangents. ISPD was measured at the vertebrae immediately above and below the fractured vertebra in each patient. This method followed the approach described by Shimizu et al. [12], which ensured consistent measurement across different observers and patient positioning [9] (Figure 1). All measurements were performed using standard lumbar spine radiographs magnified to 150%, as previously validated for cervical spine ISPD assessment [9,12]. Each ISPD value was measured twice by two independent observers at two-week intervals. Both interobserver and intraobserver reliabilities were assessed using intraclass correlation coefficients (ICCs). To account for individual variation, the difference in ISPD between standing and supine positions (ΔISPD) was normalized by dividing by the midline L4 vertebral body height on standing radiographs [13]. If L4 was fractured, L5 was used as a substitute. For easier clinical interpretation, the normalized ΔISPD value was multiplied by 100 and treated as a unitless index. Normalized ΔISPD values were calculated separately for the upper and lower adjacent levels.

Variables and definitions

The following clinical and radiographic variables were evaluated. Demographic and clinical data included age, sex, level of the fractured vertebral body, and bone mineral density (measured as the T-score of the proximal femur). Use of anti-osteoporotic medication before and after surgery was also recorded, including bisphosphonates, teriparatide, and denosumab. The primary clinical outcome was the occurrence of AVF, defined as the presence of new vertebral collapse within one year after BKP, confirmed by both clinical symptoms (recurrence of back pain) and radiological findings (loss of vertebral height and MRI signal changes). Radiographic parameters included normalized ΔISPD (upper and lower levels) [9], past history of vertebral fracture [6,14], preoperative vertebral wedge angle (≥25°) [6], postoperative correction angle (≥10°) [6], vertebral angular instability (≥14°, kyphotic angle difference) [6], preoperative local kyphosis (≥10°) [14], presence of split-type fracture [6,14,15], endplate defect ≥3 mm [6], intravertebral cleft (T2 high signal) [11], surgery timing after onset [14], and duration of back pain (≥30 days) [14].

Statistical analysis

Interobserver and intraobserver reliability for ISPD measurements was evaluated using ICCs and was classified as poor (0-0.39), moderate (0.40-0.74), or excellent (0.75-1.00). For univariate analysis, continuous variables were compared using the Mann-Whitney U test, while categorical variables were analyzed using Fisher’s exact test. Receiver operating characteristic (ROC) curve analysis was performed to determine the optimal cutoff values of normalized ΔISPD for predicting the occurrence of AVF. A p-value < 0.05 was considered statistically significant. All statistical analyses were performed using EZR software (Saitama Medical Center, Jichi Medical University, Saitama, Japan), which is a graphical user interface for R (R Foundation for Statistical Computing, Vienna, Austria).

## Results

Patient characteristics

A total of 36 patients (28 female, 8 male; mean age, 81.8 years) with OVFs at the thoracolumbar junction were included. All had ≥12 months of follow-up. Patient characteristics are shown in Table 1. Fracture levels were most frequently observed at T12 and L1 (n = 11 each), followed by L2 (n = 9), T11 (n = 4), and T10 (n = 1). With respect to osteoporosis management, preoperative treatment was administered in 9 patients, including bisphosphonates (n = 6), denosumab (n = 2), and teriparatide (n = 1). After surgery, osteoporosis treatment was initiated or continued in 19 patients, with teriparatide (n = 11) being the most common agent, followed by romosozumab (n = 6) and denosumab (n = 2).

**Table 1:** Characteristics of Patients Backgrounds (N = 36). Values are presented as mean ± SD or n (%). BMD was measured at the proximal femur. “Normalized ΔISPD” is ΔISPD/(the midline L4 vertebral body height)×100 at the upper and lower adjacent levels. Osteoporosis treatment includes bisphosphonates, denosumab, teriparatide, or romosozumab.

Characteristics	Values
Age (years)	81.8±5.8
Sex, n (female, %)	28 (77.8%)
Bone mineral density (g/cm^2^)	0.68±0.11
Preoperative osteoporosis treatment, n (%)	9 (25.0%)
Postoperative osteoporosis treatment, n (%)	19 (52.8%)
Adjacent vertebral fracture, n (%)	12 (33.3%)
Normalized upper interspinous process distance difference (ΔISPD)	14.0±10.5
Normalized lower interspinous process distance difference (ΔISPD)	19.4±16.5
Past history of vertebral fracture, n (%)	14 (38.9%)
Preoperative vertebral wedge angle (°)	18.4±6.5
Postoperative correction angle (°)	12.3±7.2
Vertebral angular instability (°)	8.9±5.9
Preoperative local kyphosis (°)	21.5±10.5
Split-type fractures, n (%)	10 (27.8%)
Endplate defects, n (%)	13 (36.1%)
Intravertebral cleft, n (%)	15 (41.7%)
Surgery timing after onset (days)	22.8±29
Duration of back pain (days)	20.4±27.4
Values are presented as the number of patients or mean ± SD	

ISPD reliability

Both interobserver and intraobserver reliabilities for ISPD were excellent (ICC: 0.882-0.913). Results are shown in Table 2.

**Table 2:** Inter‑ and intraobserver reliability of ISPD measurements. Intraclass correlation coefficients (ICCs; two‑way random effects, absolute agreement) with 95% CIs are shown by level. Measurements were repeated twice by two observers at ≥2‑week intervals on 150%‑magnified images. ISPD, interspinous process distance; ICC, intraclass correlation coefficient; CI, confidence interval.

Level	Interobserver ICC (95% CI)	Intraobserver ICC (95% CI)
T10/11	0.882 (0.811–0.933)	0.869 (0.795–0.926)
T11/12	0.901 (0.842–0.948)	0.885 (0.818–0.935)
T12/L1	0.895 (0.826–0.942)	0.873 (0.800–0.928)
L1/L2	0.913 (0.853–0.957)	0.891 (0.823–0.940)

Risk factors for AVF

AVF occurred in 12 of 36 patients (33%) within one year. Univariate analysis revealed significant differences in the AVF(+) group for normalized upper ΔISPD (p = 0.008), normalized lower ΔISPD (p = 0.015), past history of vertebral fracture (p = 0.015), preoperative vertebral wedge angle (p = 0.007), and postoperative correction angle (p ≦ 0.001) (Table 3).

**Table 3:** Univariate analysis of adjacent vertebral fracture (AVF) risk factors after balloon kyphoplasty (BKP). Continuous variables were compared using the Mann–Whitney U test; categorical variables were compared using Fisher’s exact test. Values are presented as the number of patients or mean ± SD; AVF, adjacent vertebral fracture; *P-value < 0.05 statistically significant difference.

Variables	AVF (-); N = 24	AVF (+); N = 12	P-values
Age (years）	81.2±75.9	83.1±5.7	0.82
Sex (female, %)	17 (70.8%)	11 (91.7%)	0.156
Bone mineral density (g/cm^2^)	0.68±0.21	0.64±0.06	0.659
Osteoporosis treatment intervention (%)	11 (45.8%)	8 (66.7%)	0.302
Normalized upper interspinous process distance difference (ΔISPD)	10.6±8.9	20.2±10.8	0.008*
Normalized lower interspinous process distance difference (ΔISPD)	14.4±10.2	28.4±21.8	0.015*
Past history of vertebral fracture (%)	6 (25.0%)	8 (66.7%)	0.015*
Preoperative vertebral wedge angle（≧25°, %）	2 (8.3%)	6 (50.0%)	0.007*
Postoperative correction angle（≧10°, %）	5 (20.8%)	11 (91.7%)	≦0.001*
Vertebral angular instability (≧14°, %)	17 (70.8%)	6 (50.0%)	0.281
Preoperative local kyphosis (≧10°, %)	15 (62.5%)	10 (83.3%)	0.439
Split-type fractures (%)	0 (0.0%)	1 (8.3%)	0.333
Endplate defects (%)	9 (37.5%)	4 (33.3%)	1
Intravertebral cleft (%)	18 (75.0%)	9 (75.0%)	1
Surgery timing after onset (days)	22.4±26.5	24.0±40.4	0.883
Duration of back pain (≧30days, %)	3 (12.5%)	3 (25.0%)	0.378

In contrast, no significant differences were observed between the AVF (+) and AVF (−) groups regarding age (p = 0.820), sex (p = 0.156), vertebral angular instability ≥14° (p = 0.281), preoperative local kyphosis ≥10° (p = 0.439), split-type fractures (p = 0.333), endplate defects ≥3 mm (p = 1.000), intravertebral cleft (p = 1.000), surgery timing after onset (p = 0.883), or duration of back pain ≥30 days (p = 0.378). BMD was not significantly associated with AVF occurrence (0.64 ± 0.06 vs. 0.68 ± 0.21 g/cm², p = 0.659). Likewise, osteoporosis treatment intervention before or after surgery did not differ significantly between groups (8/12, 66.7% vs. 11/24, 45.8%, p = 0.302).

ROC curve analysis

**Figure 2:**
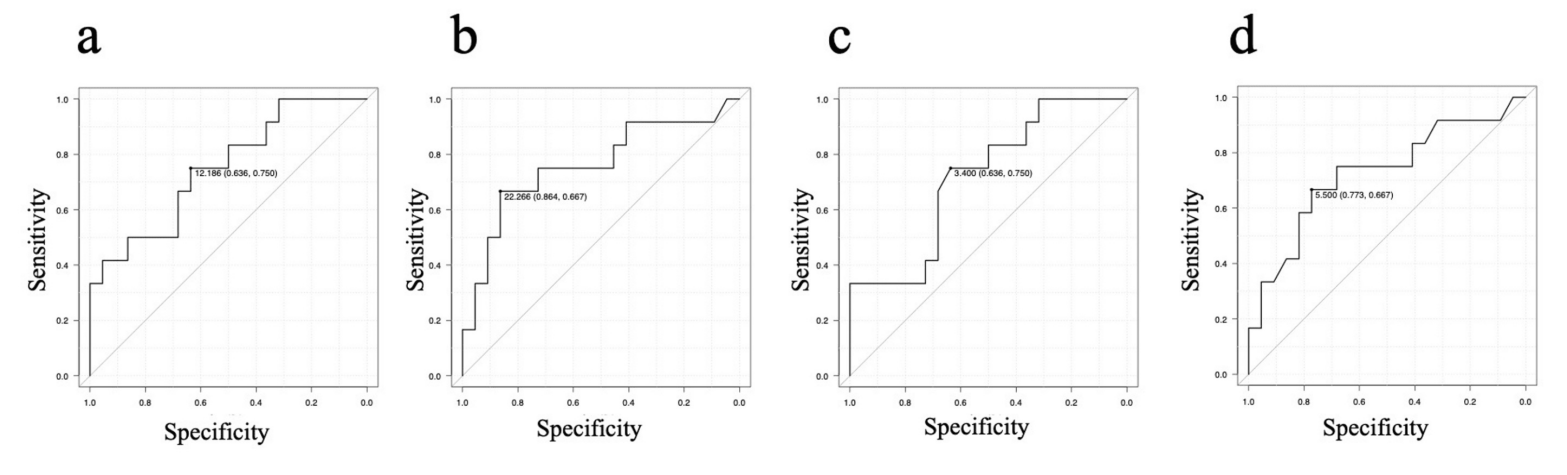
ROC curves of dynamic changes in interspinous process distance (ΔISPD) for predicting adjacent vertebral fracture (AVF) within 1 year. (a) Normalized upper ΔISPD: cutoff 12.2 (AUC 0.750; 95% CI 0.58–0.93). (b) Normalized lower ΔISPD: cutoff 22.3 (AUC 0.759; 95% CI 0.57–0.95). (c) Non‑normalized upper ΔISPD: cutoff 3.4 mm (AUC 0.718; 95% CI 0.54–0.90). (d) Non‑normalized lower ΔISPD: cutoff 5.5 mm (AUC 0.725; 95% CI 0.53–0.92). AUCs and 95% CIs are based on ROC analysis with Youden’s index.

ROC analysis of non-normalized ΔISPD revealed optimal cutoffs of 3.4 mm (upper, AUC = 0.718, 95% CI, 0.54-0.90) and 5.5 mm (lower, AUC = 0.725, 95% CI, 0.53-0.92). Normalized ΔISPD cutoffs of 12.2 (upper, AUC = 0.750, 95% CI, 0.58-0.93) and 22.3 (lower, AUC = 0.759, 95% CI, 0.57-0.95) showed comparable performance (Figure 2).

## Discussion

To our knowledge, this exploratory case series provides preliminary evidence that ΔISPD between standing and supine positions is associated with the occurrence of AVFs after BKP for OVFs. Our findings revealed that both upper and lower normalized ΔISPD values were significantly associated with AVF occurrence. The optimal thresholds were 12.2 (upper) and 22.3 (lower), corresponding to physical changes of approximately 3.4 mm and 5.5 mm, respectively. These findings suggest that posterior column laxity, captured by posture-dependent separation of the spinous processes, may be a relevant marker of AVF risk.

Our results complement prior literature that has emphasized vertebral body morphology and technical factors. Takahashi et al. developed a multivariable risk score in which greater preoperative wedge angle, larger correction angle, thoracic/thoracolumbar level, and prior OVFs increased AVF risk after BKP; these trends were directionally consistent with our univariate associations for correction angle and past history of vertebral fracture [6]. Registry data (SWISSspine) likewise linked larger preoperative segmental kyphosis to early AVF [7]. The AVA score by Hijikata et al. [14] incorporated dynamic and morphological features (instability ≥5 mm, preoperative local kyphosis ≥10°, duration of back pain ≥30 days, intravertebral cleft, and past history of vertebral fracture) and achieved an optimism-corrected AUC of 0.77 [14]. Within this context, ΔISPD adds a posterior element instability dimension that is not captured by vertebral body geometry alone and may be measurable on routine lateral radiographs. By contrast, age, BMD, and osteoporosis medications were not significant in our cohort. We attribute this to a restricted range with uniformly low BMD and limited power (12 AVF events), rather than a true null association; larger studies have shown these variables to matter under certain conditions. Accordingly, our findings should be interpreted as hypothesis-generating and complementary to, not a replacement for, established risk factors.

Mechanistically, increased ΔISPD may indicate subtle disc or ligamentous insufficiency of the posterior elements, allowing abnormal segmental motion and stress concentration at adjacent levels. This concept aligns with work in cervical pseudoarthrosis, where dynamic interspinous motion discriminates nonunion and shows high measurement reliability with 150% magnification [8,9]. Clinically, the observed ΔISPD thresholds are radiographically discernible and may help flag patients for closer follow-up or cautious perioperative decision-making. Therapeutic implications, such as when to add posterior stabilization, require prospective validation.

Limitations

This study has several limitations. First, the small single-center retrospective design limits statistical precision and precludes reliable multivariable modeling. Second, the interpretation should be considered hypothesis-generating, as the observed ΔISPD thresholds may represent sample-specific findings rather than universally applicable cutoffs. Third, ΔISPD measurements could be influenced by patient posture, pain-related guarding, degenerative changes, or radiographic quality, although we attempted to minimize these factors by using a standardized protocol. Fourth, the normalization method using the L4 anterior vertebral body height is pragmatic but nonstandard and requires external calibration and validation. Finally, the generalizability of our findings is limited to thoracolumbar OVFs treated with BKP, and prospective multicenter studies are warranted to confirm the clinical utility of ΔISPD.

## Conclusions

ΔISPD was associated with AVF occurrence in unadjusted analyses after BKP for thoracolumbar OVFs. While preliminary, these findings highlight a potential contribution of posterior element laxity to AVF risk. Given the single-center, small-event cohort and nonstandard normalization, the results should be viewed as hypothesis-generating. Prospective multicenter validation with standardized ΔISPD methodology is warranted.
